# High Total Cholesterol in Peripheral Blood Correlates with Poorer Hearing Recovery in Idiopathic Sudden Sensorineural Hearing Loss

**DOI:** 10.1371/journal.pone.0133300

**Published:** 2015-07-24

**Authors:** Nicola Quaranta, Valentina Squeo, Moris Sangineto, Giusi Graziano, Carlo Sabbà

**Affiliations:** 1 Otolaryngology, Department of Basic Medical Science, Neuroscience and Sensory Organs, University of Bari “Aldo Moro”, 70124, Bari, Italy; 2 Clinica Medica “Cesare Frugoni”, Department of Interdisciplinary Medicine, University of Bari “Aldo Moro”, 70124, Bari, Italy; 3 IRCCS Istituto Tumori Giovanni Paolo II, 70124, Bari, Italy; IRCCS Istituto Oncologico Giovanni Paolo II, ITALY

## Abstract

Idiopathic sudden sensorineural hearing loss (ISSHL) is a common otologic emergency whose cause is still unclear. The importance of blood lipids in the pathogenesis of ISSHL is widely reported in literature. In fact elevated levels of low density lipoprotein cholesterol (LDL), total cholesterol (TC) and apolipoprotein B (Apo-B) have been proposed as risk factors for this pathology. No correlation has been described between serum lipid parameters and the prognosis of ISSHL. Aim of the present study was to identify prognostic factors associated with hearing recovery in a group of patients affected by ISSHL. Ninety-four patients with the diagnosis of ISSHL hospitalized between March 2013 and October 2014 were included in this study. Patients’ blood sampling and hearing assessments were carried out. Patients were divided into two groups as “recovered” and “unrecovered”, according to their response to the treatment. We found a statistically significant higher level of total cholesterol in the unrecovered group compared to the recovered one (p = 0.03). None of the other routine laboratory parameters have shown a statistically significant difference between the patients successfully treated and patients with poor outcomes. Total cholesterol concentrations may be a prognostic factor for recovery in ISSHL and should be assessed together with routine tests in patients with this condition. The other routine laboratory parameters seem to have no effect on the development and prognosis of this pathology.

## Introduction

Idiopathic Sudden Sensorineural Hearing Loss (ISSHL) is commonly defined as a hearing loss of at least 30 dB over 3 contiguous test frequencies occurring within a 72-h period [1]. The estimated incidence of ISSHL is approximately 10/100,000 person per year, with no differences in gender and side affected [2].

The aetiology of ISSHL is idiopathic in most of the cases [3]. Recent studies have however highlighted the role of microcirculatory disturbances in the pathogenesis of this disease. This hypothesis has been supported by findings of abnormal red cell filterability [4], and increased plasmatic and whole blood viscosity [5] in ISSHL patients. Rudack et al. [6] have reported that elevated fibrinogen levels, smoking and the glycoprotein Ia C807T polymorphism are associated with an increased risk for this syndrome. Our group has previously reported that patients affected by ISSHL present higher serum levels of circulating adhesion molecules (sICAM-1 and sVCAM-1) [7], a reduced percentage of circulating endothelial progenitor cells [8] and an altered flow-mediated-dilatation (FMD) [9] as clear early markers of endothelial dysfunction.

From a clinical point of view, the prognosis of ISSHL declines in patients with higher age, worse initial hearing level, longer time from onset to treatment, and presence of vertigo [10]. Using these prognostic factors Suzuki *et al*. [11] formulated a multiple regression equation that was able to predict hearing recovery rate with 70% probability. Kanzaki *et al*. correlated hearing recovery in patient affected by ISSHL with peripheral blood indices [12]; they found that high fibrinogen level, high white cells count (WBC), and high erythrocyte sedimentation rate (ESR) correlated with poorer recovery when patients were treated in the first week from onset of hearing loss.

Aim of the present study was to identify prognostic factors associated with hearing recovery in a group of patients affected by ISSHL.

## Materials and Methods

The study group included 94 subjects affected by unilateral ISSHL consecutively hospitalized between March 2013 and October 2014. Inclusion criteria for this study were: hearing loss of >30 dB hearing level (HL) affecting at least three contiguous frequencies occurring in less than 72 hours; normal hearing in the contralateral ear (air conduction pure-tone average (PTA) for the frequencies 0.25, 0.5, 1, 2, 3, 4, and 8 kHz < 40 dB HL); negative history of hearing loss or ear surgery in the affected ear; no impairment of cranial nerves; negative magnetic resonance imaging (MRI) with gadolinium for VIII cranial nerve pathology.

All patients affected by Meniere’s disease, herpes zoster oticus, noise-induced hearing loss, and other known causes of inner ear disease were excluded.

In all patients age, gender, height (cm), weight (kg), blood pressure, body mass index (BMI) (kg/m2), smoking behaviour (yes/no), presence of cardiovascular risk factors (1 of the following diseases/history of diseases: vein thrombosis, cardiac infarction, diabetes mellitus, hypertension, hyperlipidaemia), tinnitus (yes/no), vertigo or dizziness were recorded.

In all subjects a standard audiovestibular investigation was carried out. It consisted of pure-tone and speech audiometry, impedance audiometry and bi-thermal caloric testing of the vestibular function. PTA was calculated from the air conduction thresholds at 0.25, 0.5, 1, 2, 3, 4, and 8 kHz. Pure tone and speech audiometry were tested every 48h until hospital discharge.

Peripheral blood samples for chemistry and hematologic tests were collected from the patients. The assessment of pre-treatment laboratory values included: hematologic profile with white blood cells, neutrophil, lymphocyte and platelets; glucose; ESR; total cholesterol, LDL, high density lipoprotein (HDL), triglycerides, pro-thrombin time (PT-INR), and fibrinogen. Blood samples were taken before starting the treatment to rule out any related perturbance on the laboratory values.

All patients were treated with standard ISSHL protocol, which included carbogen (95% CO2 and 5% O2) inhalation, pentoxifylline, vitamin C, magnesium and oral steroids (prednisone at 1 mg/kg per day). Patients who required specific therapy for diabetes, hypertension or dyslipidaemia were treated in association with the standard therapy.

Recovery was evaluated as follows:

(initial PTA—final PTA of the affected ear)/(initial PTA of the affected ear-PTA of the opposite ear) x100 [13].

Full recovery was defined as improvement greater than 75%, an improvement rate between 46 and 75% was defined as good, between 20 and 45% as fair and less than 20% as no improvement.

All patients signed an informed consent and the work was performed in accordance with the principles of the 1983 Declaration of Helsinki. The study has been executed according to the normal clinical practice guidelines and the analysis was ex-post on data that do not interfere with patients’ privacy. All the data were entered in a computerized database and were anonymized and de-identified prior to analysis. The “Comitato Etico Azienda Ospedaliero Universitaria Policlinico di Bari” was therefore informed and provided approval.

Baseline characteristics of the study population were calculated and results were expressed as mean ± SD for continuous variables and as frequencies and percentages for categorical variables. Comparisons of clinical parameters between the groups of interest (low and high recovering percentage) were performed with T-test and the Pearson χ^2^ test or Fisher’s Exact test, as appropriate, for continuous and categorical variables, respectively. Paired t-test was also used to assess pre-post comparison between tonal and speech audiometry parameters in recovered and unrecovered group. Pearson’s correlation was used when appropriate. The statistical significance was achieved at a p-value<0.05. All the analyses were performed using the Statistical Analysis System (SAS Institute, Cary, NC) software.

## Results

Ninety-four patients were included in this study (S1 Table). Demographic and clinical characteristics of the study population with normal reference values for all laboratory parameters are given in Table 1. The associated diseases and clinical presentation are reported in Table 2.

**Table 1:** Demographic and clinical characteristics of the study population.

Parameter	Mean	SD	Min	Max
Age (years)	48.4	16.7	13	83
Height (m)	1.7	0.1	1.5	1.9
*Weight (kg)*	73.8	15.4	38	135
*BMI (kg/m2)*	25.3	4.5	15.6	37.4
*Systolic PA*	119.7	13.4	90	150
*Diastolic PA*	76.2	6.6	60	90
*Fasting glucose (mg/dl)*	97.8	30.4	53	241
*Cholesterol (mg/dl)*	184.8	42.7	96	289
*HDL-C (mg/dl)*	53.4	14.3	2	86
*LDL-C (mg/dl)*	110	37.5	34	219
*Triglycerides (mg/dl)*	101.8	54	19	340
*PT-INR*	1.2	0.7	0.9	5.9
*Fibrinogen (mg/dl)*	257	52.5	161	446
*HCT (%)*	41.7	4	30.2	51.2
*Neutrophil (103/u)*	5.5	2.2	2	12.1
*Lymphocyte (103/u)*	2.1	0.9	0.2	4.8
*NLR*	3.7	5.9	0.6	54.1
*Platelet (103/u)*	249.5	56.2	135	402
*WBC (103/u)*	8.2	2.4	3.3	16.5
*PLR*	157.8	185.5	27	1736.8
*ESR (mm/h)*	11.5	9.7	2	46

Demographic and clinical characteristics of the study population (n = 94). BMI: body mass index; ESR: erythrocyte sedimentation rate; HCT haematocrit; HDL-C: high density lipoprotein cholesterol; LDL-C: low density lipoprotein cholesterol; NLR neutrophil to lymphocyte ratio; PA: arterial pressure; PLR platelets to lymphocyte ratio; PT-INR pro-thrombin international normalised ratio; WBC White blood cells.

**Table 2:** Symptoms and associated diseases in study population.

	YES	NO
**Smoking**	25 (27%)	66 (73%)
**Hypertension**	35 (39%)	56 (61%)
**Hyperglicemia**	31 (33%)	62 (67%)
**TAG > 150MG/DL**	15(17%)	74 (83%)
**PATHOLOGICAL HDL**	19 (22%)	68 (78%)
**Tinnitus**	75 (80%)	19 (20%)
**Vertigo**	21 (26%)	70 (74%)
**Fullness**	60(64%)	34 (35%)

F: female; M: male; HDL: High density lipoprotein; TAG: Triglycerides.

Mean PTA before treatment was 57.52±25.11 dB HL, mean PTA at hospital discharge was 39.92±26.91 dB HL and mean PTA on the normal ear was 21,46±12,43 dB HL. As for the speech audiometry the mean SRT of the affected ear at admission was 63.93±37.95 dB SPL and 47±36.6 dB SPL at discharge, mean SDS was 65,65±38,64% at admission and 78,73±34,31% at discharge. 41% of our patients showed complete recovery, 13.3% a good recovery, 20% a fair recovery and 25.5% had no improvement.

**Fig 1:**
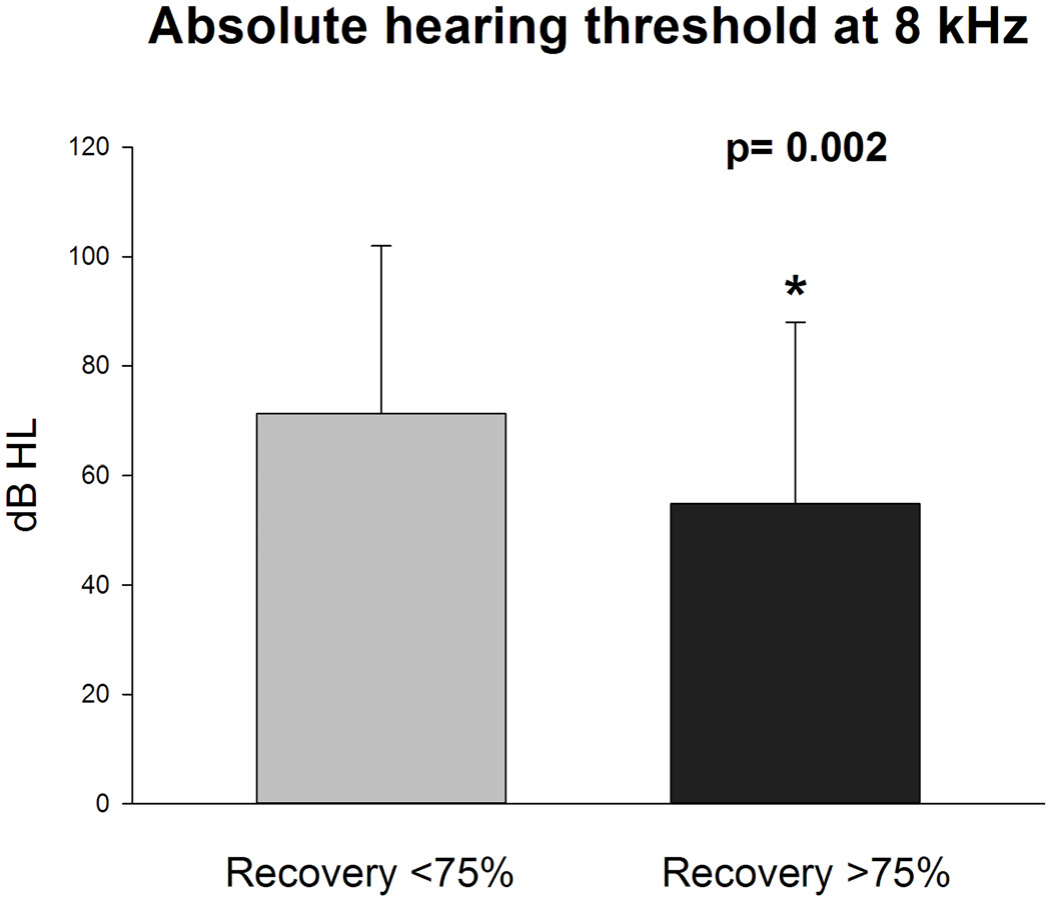
Higher cholesterol levels correlate with lower recovery rates. Pearson’s correlation (P = 0.03; R = -0.2).

Statistical analysis was performed according to the recovery rate. Correlation analysis showed a significant negative correlation between recovery and total cholesterol levels (R -0,233; p 0.034) (Fig 1). Interestingly, recovery was positively correlated with contralateral PTA (R 0,22; p 0,04). All the other blood indices were not correlated with recovery.

**Fig 2:**
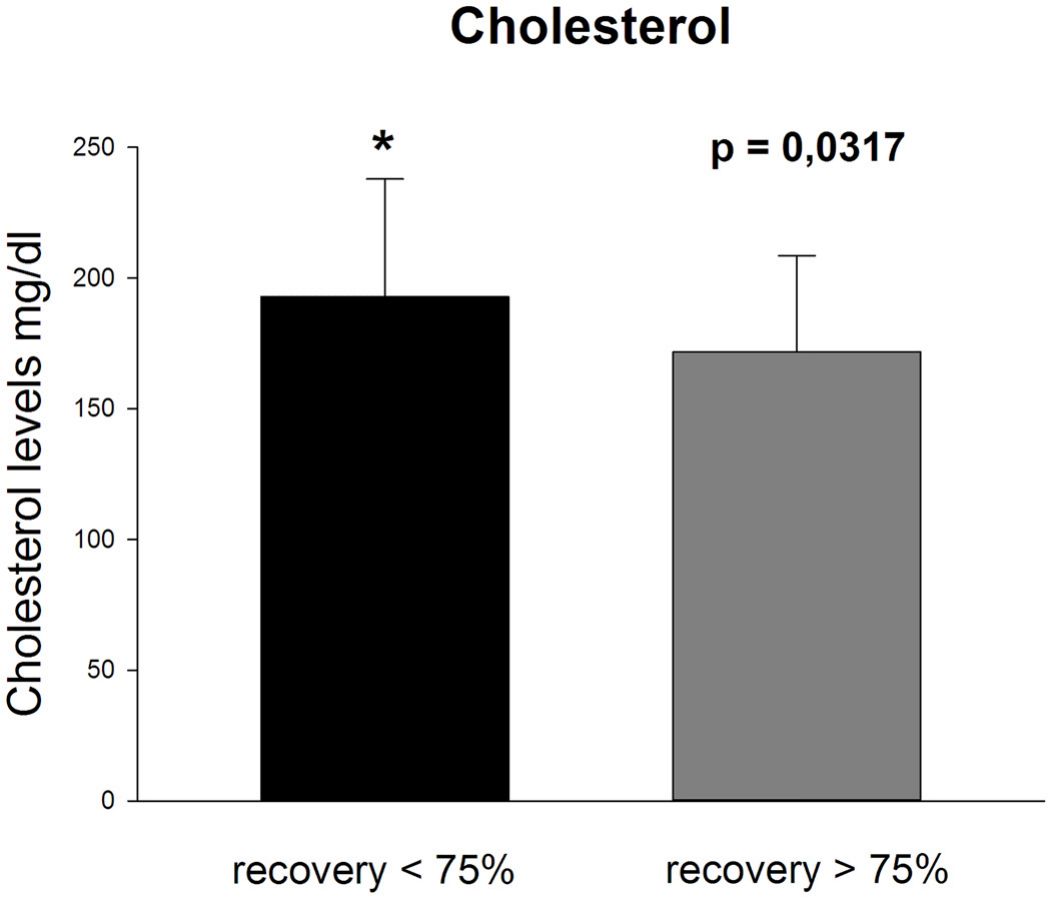
The unrecovered group presents higher total cholesterol levels than recovering group. Data are represented as mean±SD; significantly different recovery <75% versus recovery >75% (P = 0.0317).

**Fig 3:**
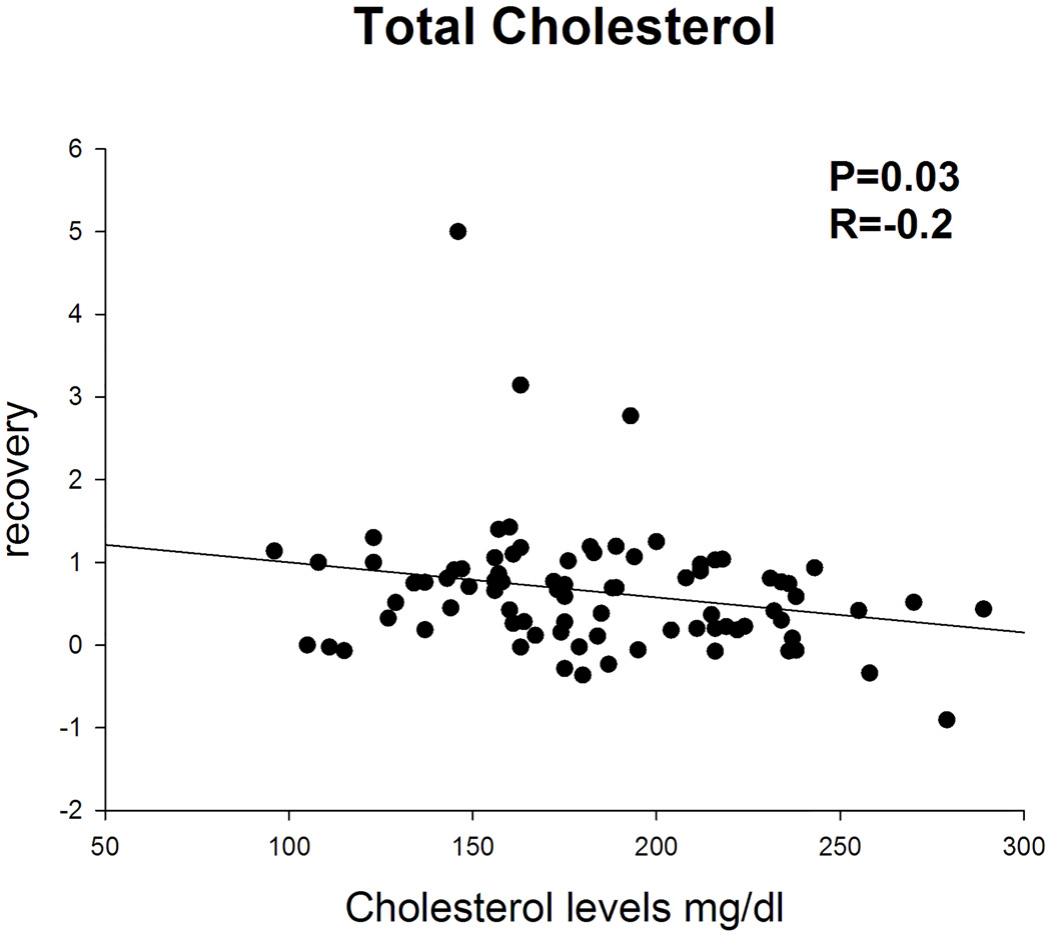
Absolute hearing threshold at 8 kHz at admission is lower in recovering patients. Data are represented as mean±SD; significantly different recovery >75% versus recovery <75% (P = 0.002).

Prognostic factors were evaluated in patients showing a recovery greater than 45% (good and complete) and greater than 75% (complete). In the first case (>45% recovery) statistical analysis showed a trend towards lower values of total cholesterol (p 0,0584) in patients showing recovery. No differences were found for all the other clinical and blood indices. In patients showing complete recovery (>75%) total cholesterol was significantly lower compared to the rest of the subjects (p 0,03) (Fig 2) (Table 3). Absolute hearing threshold at 8 kHz was significantly lower in patients recovering (71,25 ± 30,73 dB HL versus 54,86±33,16 dB HL; p 0,02) (Fig 3), no significant differences were found in terms of hearing threshold both on the affected and non-affected ear.

**Table 3:** Recovered group vs unrecovered group.

Variable	Rec > 0.75%	Rec < 75%	p value
*Age (years)*	46.3±18.4	48.1±16.0	0.6045
*Height (m)*	1.7±0.1	1.7±0.1	0.3951
*Weight (kg)*	73.1±18.1	73.8±13.3	0.8407
*BMI (kg/m2)*	24.6±4.7	25.6±4.3	0.4333
*Systolic PA (mmHg)*	117.1±13.8	120.3±12.2	0.2705
*Diastolic PA (mmHg)*	75.0±7.0	76.6±5.6	0.2953
*Fasting glucose (mg/dl)*	99.3±35.7	92.2±17.5	0.8463
*Cholesterol (mg/dl)*	171.7±36.8	192.8±45.1	0.0317*
*HDL-C (mg/dl)*	52.4±13.6	54.2±15.1	0.5606
*LDL-C (mg/dl)*	101.3±33.1	114.8±39.0	0.1206
*Triglycerides (mg/dl)*	90.7±37.6	109.2±63.0	0.3655
*PT-INR*	1.1±0.5	1.2±0.8	0.8377
*Fibrinogen (mg/dl)*	267.9±63.1	248.4±45.4	0.2264
*HCT (%)*	41.7±4.7	41.7±3.7	0.8296
*WBC*	8.0±2.0	8.5±2.7	0.4955
*Neutrophils*	5.5±1.8	5.7±2.4	0.9817
*Lymphocytes*	2.0±0.9	2.2±0.9	0.3467
*Platelet*	254.3±54.9	247.6±60.3	0.7051
*NLR*	3.5±3.0	4.0±7.7	0.6736
*PLR*	158.1±96.5	161.0±243.4	0.0901
*ESR (mm/h)*	12.3±10.4	10.8±9.8	0.4881
*PTA AE ad (dB HL)*	51.7±20.2	57.9±25.1	0.3475
*PTA NAE ad (dB HL)*	22.2±12.0	19.6±11.5	0.2028
*PTA AE dis (dB HL)*	21.9±11.3	48.7±24.4	<0.0001*
*Recovery rate*	1.2±0.8	0.2±0.3	<0.0001*

Clinical characteristics, blood and instrumental parameters of the recovered group and unrecovered group. ad: admission; AE: affected ear; BMI: body mass index; dis: discharge; ESR: erythrocyte sedimentation rate; HCT: haematocrit; HDL-C: high density lipoprotein cholesterol; LDL-C: low density lipoprotein cholesterol; NAE: non affected ear; NLR: neutrophil to lymphocyte ratio; PLR: platelets to lymphocyte ratio; PT-INR: pro-thrombin international normalised ratio; PTA: pure-tone average; WBC: White blood cells.

## Discussion

The results of our study show that lower TC was associated with better recovery in patients affected by ISSHL.

The aetiology of ISSHL is still unclear and there are different hypothesis about risk factors for this condition [3]. We have previously demonstrated an increased expression of adhesion molecules, a reduced percentage of circulating endothelial progenitor cells and an altered FMD in ISSHL patients as early signs of endothelial damage [7,8,9]. We have hypothesized that the endothelial dysfunction could predispose to the development of a pro-thrombotic state at the level of the inner ear and considering that the cochlear vascular tree is a terminal type, an interruption of vascular flow due to endothelial damage impairs cochlear membrane functions. Other authors have reported the overlap between risk factors for ISSHL and cardiovascular disease supporting a vascular alteration at the base of ISSHL [6, 14, 15, 16]. Among all cardiovascular risk factors the role of dyslipidaemia in the pathogenesis of ISSNHL is debated. A clinical study of 250 Chinese subjects showed that the levels of TC, LDL-C and Apo-B were significantly higher in patients with ISSNHL than in control subjects [17]. Cadoni et al [18] in patients affected by ISSHL reported that hypercholesterolemia and saturated fatty acid, together with low levels of coenzyme Q10 and nervonic acid, were associated with an elevated risk of ISSHL. However, a recent systematic review and meta-analysis identified only 6 case-control study that did not provide evidence for serum lipids associated with SSHL, nor ruled out such association [19].

From a clinical point of view the prognosis of ISSHL declines in patients with higher age, worse initial hearing level, longer time from onset to treatment, and presence of vertigo [10]. Kanzaki *et al*. reported that high fibrinogen levels, high WBC counts and high LDL values correlate with poorer hearing recovery in ISSHL [12].

High fibrinogen levels are associated with high blood viscosity and may indicate ischemic changes in the inner ear [12, 20], while WBC count is a classic inflammatory marker especially in cardiovascular diseases [21]. Within WBC, neutrophil to lymphocyte ratio (NLR) has been also described as an easily measurable indicator of systemic inflammation [22]. NLR increases with age and has been reported as an independent prognostic factor for diabetes, chronic kidney disease, heart failure, hypertension and coronary artery disease [22]. Recently, NLR levels have been reported to be significantly higher in patients with ISSNHL and to correlate with poor recovery [23].

In the present study we couldn’t find a significant correlation between fibrinogen, WBC and NLR with hearing recovery, while TC level was the only prognostic factor. The way high cholesterol interferes with hearing recovery has never been investigated. It is, however, well known that high levels of TC are associated with an increased pro-thrombotic state [24]; in addition studies on Apo-E knockout mice showed that hyperlipidaemia and atherosclerosis are associated with hearing impairment and histological findings of spiral modiolar artery stenosis and thickening of the vascular intima [25]. Hyperlipidaemia and saturated fatty acid have been also correlated with oxidative stress mechanisms [18] that may play a role not only in noise induced hearing loss and ototoxicity, but also in the pathogenesis of ISSHL.

It is well known that higher levels of LDL cholesterol are associated with higher mortality [26,27], in fact the last guidelines for the treatment of blood cholesterol of the 2013 from American College of Cardiology/American Heart Association (ACC/AHA) encourage adherence to a healthy lifestyle (diet and physical activity), control of blood pressure and diabetes, and avoidance of smoking for all adults. Statin therapy is recommended in patients with clinical atherosclerotic cardiovascular disease (ASCVD), or in primary prevention for adults with LDL level ≥ 190 mg/dl, those aged 40 to 75 years with diabetes, and those with a 10-year ASCVD risk ≥7.5% without diabetes [28].

## Conclusions

This retrospective study involving 94 ISSNHL patients indicates total cholesterol concentrations may be a prognostic factor for recovery in ISSHL, and should be assessed during the investigation of patients with this condition. High cholesterol levels, even if they are not associated with a higher risk of developing ISSHL, could influence hearing prognosis in these patients and should be kept as low as possible as suggested by ACC/AHA.

## Supporting Information

S1 Table Database sudden HL. Gender: 0 = female, 1 = male; Affected side: 0 = left, 1 = right; N/L: ratio neutrophils/lymphocytes; P/L: ratio platelets/lymphocytes; For the remaining columns: 0 = no; 1 = yes; The columns AA, AB, AC, AD, AE describe the positivity criteria of metabolic syndrome according to ATP III: WC = waist circumference criterion, HYP = hypertension criterion, GLC = hyperglycemia criterion, TG = hypertrygliceridemia criterion, HDL = low HDL criterion; From the column AS to the column AY there are auditory thresholds (dB) of the affected ear at different frequencies; SRT_Db_AE: Speech Reception Threshold (dB) of the affected ear; SDS_AE: Speech Discrimination Score (%) of the affected ear; From the column BB to the column BH there are auditory thresholds (dB) of the not affected ear at different frequencies; SRT_dB_NAE: Speech Reception Threshold (dB) of the not affected ear; SDS_AE: Speech Discrimination Score (%) of the not affected ear; from the column BR to the column BZ there are described auditory thresholds (dB), the SRT and the SDS of the affected ear at discharge; PTA: Pure-tone average; AE: affected ear; NAE: not affected ear.
(XLSX)
