# Correction: High Total Cholesterol in Peripheral Blood Correlates with Poorer Hearing Recovery in Idiopathic Sudden Sensorineural Hearing Loss

**DOI:** 10.1371/journal.pone.0138845

**Published:** 2015-09-18

**Authors:** The PLOS ONE Staff

The images for Figs 1 and 3 are incorrectly switched. The image that appears as Fig 1 should be Fig 3, and the image that appears as Fig 3 should be Fig 1. The figure captions appear in the correct order.

**Fig 1:**
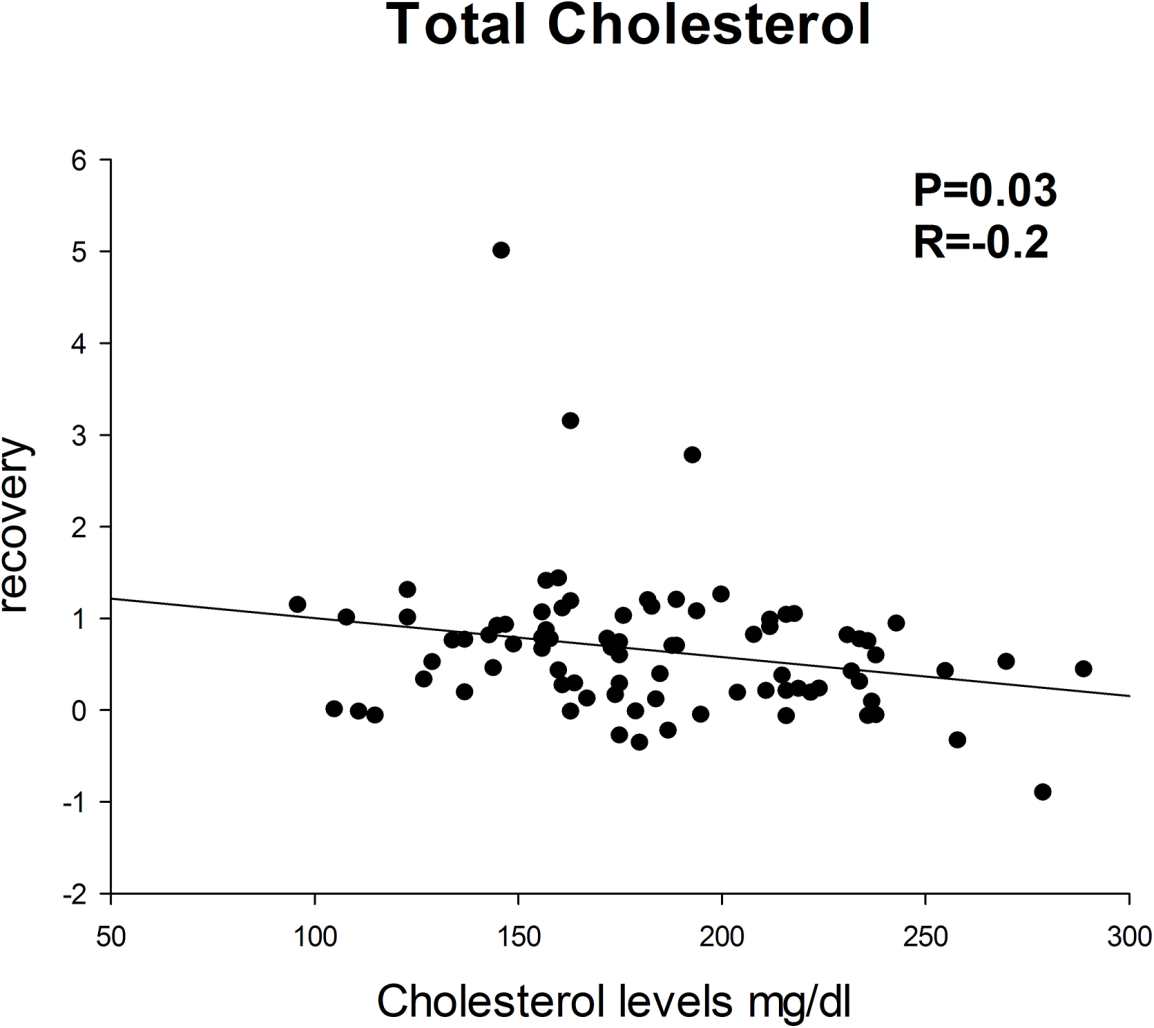
Higher cholesterol levels correlate with lower recovery rates. Pearson’s correlation (P = 0.03; R = -0.2).

**Fig 3:**
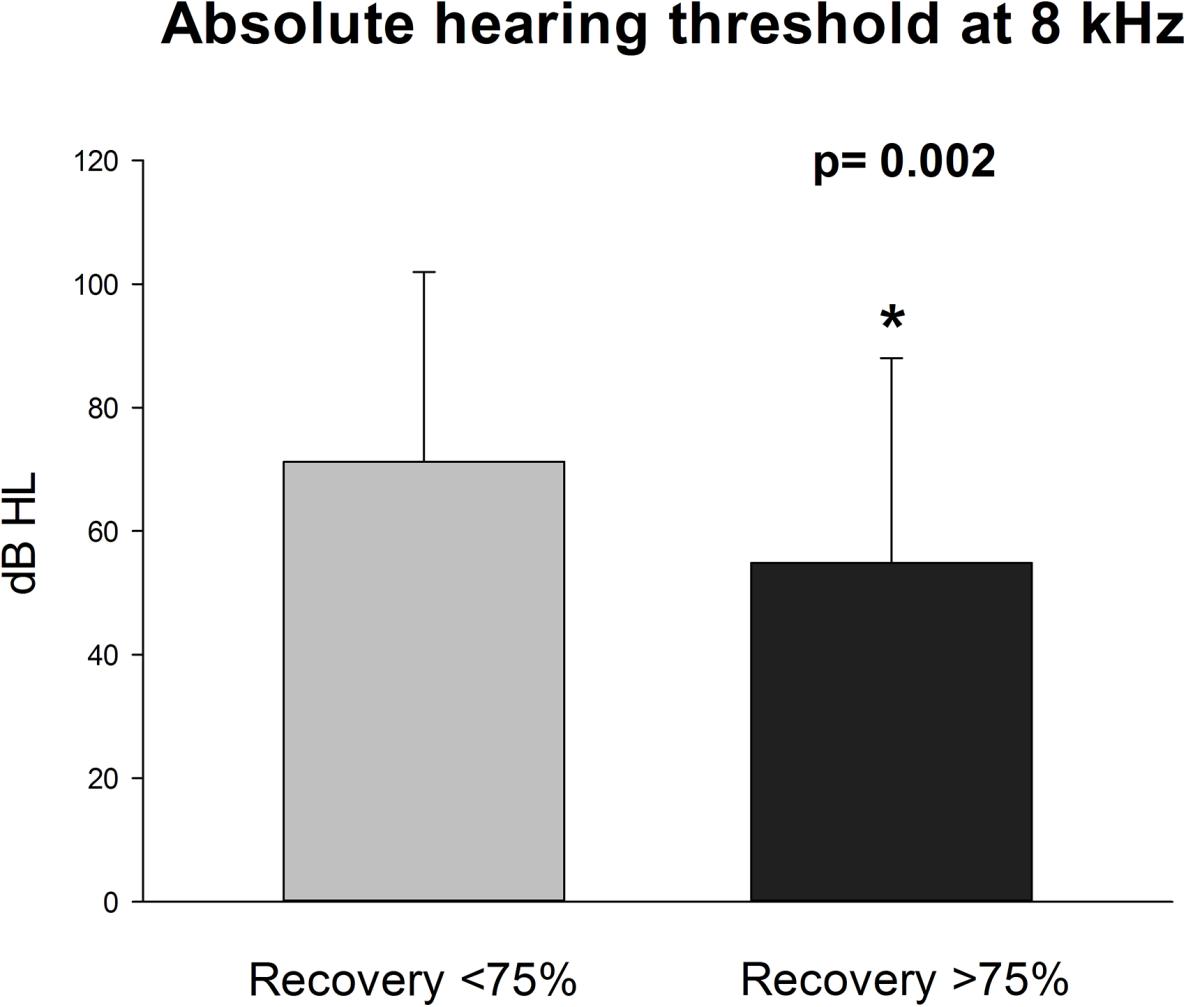
Absolute hearing threshold at 8 kHz at admission is lower in recovering patients. Data are represented as mean±SD; significantly different recovery >75% versus recovery <75% (P = 0.002).
